# Myricitrin Alleviates Oxidative Stress-induced Inflammation and Apoptosis and Protects Mice against Diabetic Cardiomyopathy

**DOI:** 10.1038/srep44239

**Published:** 2017-03-13

**Authors:** Bin Zhang, Qiang Shen, Yaping Chen, Ruile Pan, Shihuan Kuang, Guiyan Liu, Guibo Sun, Xiaobo Sun

**Affiliations:** 1 Institute of Medicinal Plant Development, Peking Union Medical College and Chinese Academy of Medical Sciences, Beijing 100193, China; 2 Key Laboratory of Bioactive Substances and Resources Utilization of Chinese Herbal Medicine, Ministry of Education, Beijing 100193, China; 3 Beijing Key Laboratory of Innovative Drug Discovery of Traditional Chinese Medicine (Natural Medicine) and Translational Medicine, Beijing 100193, China; 4 Key Laboratory of efficacy evaluation of Chinese Medicine against glyeolipid metabolism disorder disease, State Administration of Traditional Chinese Medicine, Beijing 100193, China; 5 Center of Research and Development on Life Sciences and Environmental Sciences, Harbin University of Commerce, Harbin 150076, China; 6 School of Life Science, Beijing Institute of Technology, Beijing 100081, China; 7 Department of Animal Sciences, Purdue University, West Lafayette, IN 47907, USA

## Abstract

Diabetic cardiomyopathy (DCM) has been increasingly considered as a main cause of heart failure and death in diabetic patients. At present, no effective treatment exists to prevent its development. In the present study, we describe the potential protective effects and mechanisms of myricitrin (Myr) on the cardiac function of streptozotosin-induced diabetic mice and on advanced glycation end products (AGEs)-induced H9c2 cardiomyocytes. *In vitro* experiments revealed that pretreatment with Myr significantly decreased AGEs-induced inflammatory cytokine expression, limited an increase in ROS levels, and reduced cell apoptosis, fibrosis, and hypertrophy in H9c2 cells. These effects are correlated with Nrf2 activation and NF-κB inhibition. *In vivo* investigation demonstrated that oral administration of Myr at 300 mg/kg/day for 8 weeks remarkably decreased the expression of enzymes associated with cardiomyopathy, as well as the expression of inflammatory cytokines and apoptotic proteins. Finally, Myr improved diastolic dysfunction and attenuated histological abnormalities. Mechanistically, Myr attenuated diabetes-induced Nrf2 inhibition via the regulation of Akt and ERK phosphorylation in the diabetic heart. Collectively, these results strongly indicate that Myr exerts cardioprotective effects against DCM through the blockage of inflammation, oxidative stress, and apoptosis. This suggests that Myr might be a potential therapeutic agent for the treatment of DCM.

Diabetes mellitus (DM) has become a global public health issue. The total number of adults worldwide experiencing DM was 415 million in 2015, and is estimated to increase to 642 million by 2040^1^. Substantial progress has been made in the treatment of diabetes in recent years. However, effective strategies to fight against its complications remain limited. Among the complications induced by DM, DCM is a leading cause of morbidity and mortality^2^. Epidemiological studies have shown that diabetic patients are exposed to a 2- to 5-fold increase risk in developing cardiac abnormalities compared with age-matched non-diabetics. Pathologically, diabetes induces cardiac dysfunction with no evidence of hypertension and coronary artery disease, and is characterized by myocardial insulin resistance, cardiac fibrosis, ventricular hypertrophy and heart failure^3^.There are currently no effective approaches to treat DCM in the clinic. Thus, it is of great importance to clarify its molecular mechanisms and to search for potential compounds to provide protection against DCM.

Multiple mechanisms have been proposed to contribute to the pathophysiology of DCM. These include oxidative stress, inflammation, disordered calcium handling, mitochondrial dysfunction and fibrosis^4^,^5^,^6^,^7^,^8^. It has been demonstrated that AGEs are implicated in the pathogenesis of diabetes and represent a risk factor for its complications^9^,^10^,^11^. Additionally, high levels of AGEs appear to be tightly related to instances of DCM, which result in superfluous oxidative stress and inflammatory responses. Ko and his colleagues found that 400 μg/ml AGEs could increase ROS production through suppression of antioxidant Nrf-2 and downstream pathway in H9c2 cells^12^. Clinically, serum AGEs levels were increased not only in patients with osteoporosis (14.75 *vs.* 8.12 U/ml)^13^, but in DM patients with vascular complications (3.40 *vs.* 1.12 μg/ml)^14^, indicating that AGEs are associated with cardiac injury resulting from DCM. Both oxidative stress, and inflammation directly triggered by persistent high glucose or AGEs, may occur as upstream events. Crosstalk between oxidative stress and inflammation signaling can feed back to further disturb each other^15^. Thus, to inhibit oxidative stress and inflammation simultaneously seems to be a favorable strategy for the treatment of DCM.

Myricitrin (Fig. 1A) is an important flavone distributed in the root bark of *Myrica cerifera, Myrica esculenta, Ampelopsis grossedentata* and other plants. Our group previously found that Myr could prevent endothelial cell apoptosis by activating PI3K/Akt signaling. It was further verified in our laboratory that Myr could also suppress H9c2 cell apoptosis induced by high glucose^16^. In addition, Myr was reported to possess anti-inflammatory and anti-fibrotic properties^17^. Nevertheless, the protective effects of myricitrin on DCM has not yet been investigated.

In this study, we investigated the potential protective role of myricitrin in diabetic hearts and in H9c2 cardiomyocytes exposed to AGEs. We found that Myr effectively ameliorated the cardiac dysfunction of diabetic mice and prevented cardiomyocyte apoptosis by up-regulating Nrf2, and down-regulating NF-κB signaling.

## Results

### Myricitrin protects H9c2 cardiomyocytes from AGEs-induced cell death

**Figure 1:**
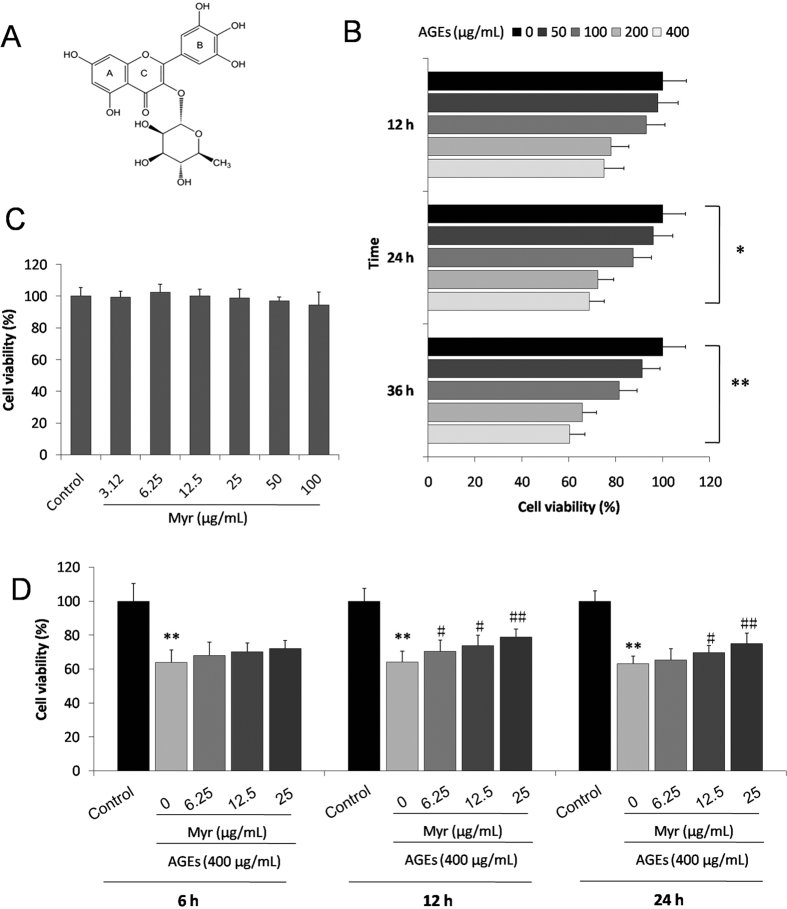
Myr attenuated H9c2 cell death induced by AGEs. (**A**) Chemical structure of myricitrin. (**B**) Cell viability was assessed by the MTT assay. H9c2 cells were exposed to various concentrations of AGEs for 12, 24, and 36 h in the presence or absence (control) of myricitrin (**C**) The toxic effect of myricitrin on H9c2 cell viability was observed. (**D**) The protective effects of myricitrin on H9c2 cells exposed to AGEs (400 μg/mL). Values are represented as the mean ± SD. n = 9, *p < 0.05 and **p < 0.01 vs control group; ^#^*p* < 0.05 and ^##^*p* < 0.01 vs AGEs group.

To investigate the protective effects of Myr on AGEs-induced H9c2 cardiomyocyte death, we initially evaluated the general toxicity of AGEs. The H9c2 cells were incubated with a series of AGEs concentrations (0, 50, 100, 200, and 400 μg/ml) for 12, 24, and 36 h, respectively. An MTT assay was employed to measure cell viability. As shown in Fig. 1B, cell viability was decreased to 60.34 ± 6.52% when treated with 400 μg/ml AGEs for 36 h. Thus, this study used 400 μg/ml AGEs in subsequent experiments. The cytotoxic effect of the agent on H9c2 cells was simultaneously measured. After treatment with various doses (3.12–100 μg/ml) of Myr for 24 h, no significant change in cell viability was observed (Fig. 1C). Subsequently, the potential cardioprotective effects of Myr against AGEs-induced H9c2 cardiomyocytes injury were assessed. As shown in Fig. 1D, cell viability increased to 70.52 ± 6.51%, 73.67 ± 5.30%, and 78.94 ± 4.52% with pretreatment with different doses (6.25. 12.5 and 25 μg/ml, respectively) of Myr for 12 h compared with exposure to only 400 μg/ml AGEs for 36 h. The optimal concentration and pretreatment time with Myr was 25 μg/ml and 12 h respectively. These results indicated that Myr could prevent H9c2 cardiomyocytes from AGEs-induced cell death.

### Myricitrin inhibits AGEs-induced inflammation and oxidative stress in H9c2 cells

**Figure 2:**
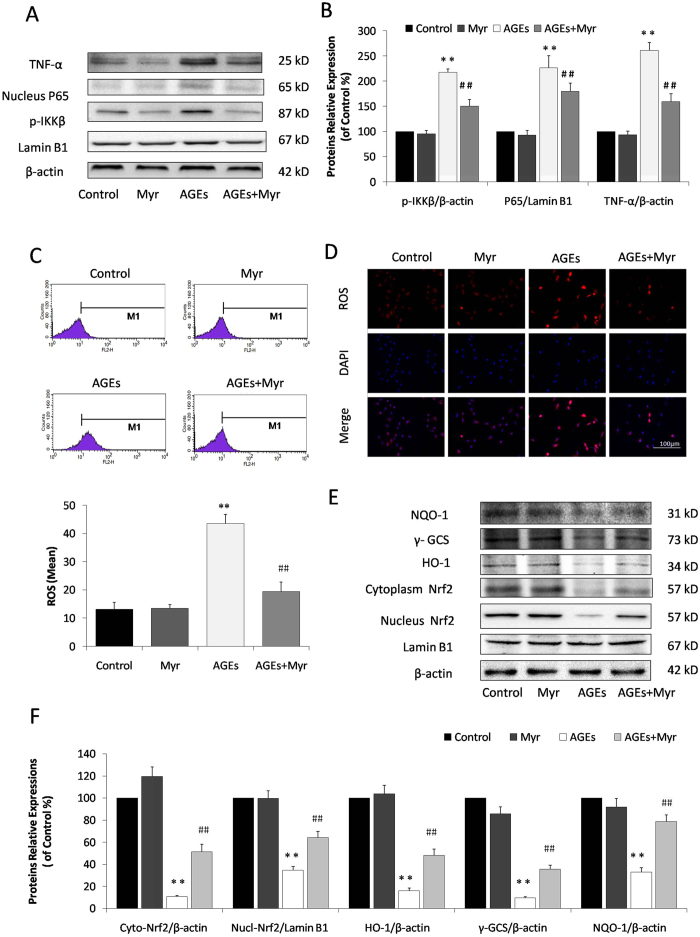
Myr inhibited AGEs-induced inflammation and oxidative stress. (**A**) The expression of NF-κB signaling and TNF-α in H9c2 cells by immunoblotting analysis. (**B**) The relative expression levels of p-IKKβ, nuclear P65, and TNF-α in relation to β-actin were expressed in the bar graphs. (**C**) Intracellular ROS levels in H9c2 cardiomyocytes evaluated using a flow cytometer. (**D**) Representive images of ROS staining. Cells with red influorescence indicated elevated intracellular ROS level. (**E**) Immunoblotting analysis of Nrf2-mediated anti-oxidative enzymes in H9c2 cells. (**F**) The relative protein expression of Cyto-Nrf2, Nucl-Nrf2, HO-1, γ-GCS, and NQO-1 to β-actin were expressed in the bar graphs. n = 3, **p < 0.01 vs the control group; ^##^*p* < 0.01 vs AGEs group.

Previous study has demonstrated inflammation in diabetes, which features NF-κB activation induced by AGEs^18^. Thus, we examined the effects of Myr on NF-κB signaling and the inflammatory cytokine, TNF-α. Incubation with AGEs for 36 h in H9c2 cells significantly promoted TNF-α and phospho-IKK-β expression, and the translocation of P65 from cytosol to the nucleus. These alterations were markedly reduced by the pretreatment with Myr at 25 μg/ml (Fig. 2A,B).

Besides inflammation, oxidative stress is a key mechanism involved in the pathogenesis of cardiovascular disease in diabetes. The above two can be concurrently induced by excess AGEs^19^. Intracellular ROS levels were assessed by measuring mitochondrial superoxide using MitoSOX Red. Compared with the control, AGEs treatment significantly increased ROS production in H9c2 cells by almost 3-fold. However, Myr preconditioning markedly reduced this increase (Fig. 2C,D, *p* < 0.01).

Many anti-oxidant genes, such as heme oxygenase-1 (HO-1) and quinine oxidoreductase 1 (NQO-1), both of which are regulated by the Nrf2 signaling, have been implicated in fighting against oxidative stress. We examined the impact of Myr on Nrf2-regulated antioxidant enzymes. Western blot analysis showed that AGEs significantly down-regulated Nrf2 expression and inhibited its translocation from the cytosol to the nucleus, and that this could be reversed by the pretreatment of H9c2 cells with Myr (Fig. 2E,F, *p* < 0.01). Consistent with Nrf2 changes, Myr markedly attenuated the inhibition of NQO-1, γ-GCS, and HO-1 expression induced by AGEs (*p* < 0.01). These results collectively demonstrate that Myr plays potent cardioprotective roles in the suppression of inflammation and oxidative stress via the regulation of NF-κB and Nrf2 signaling.

### Myricitrin attenuates AGEs-induced mitochondrial injury and apoptosis in H9c2 cells

**Figure 3:**
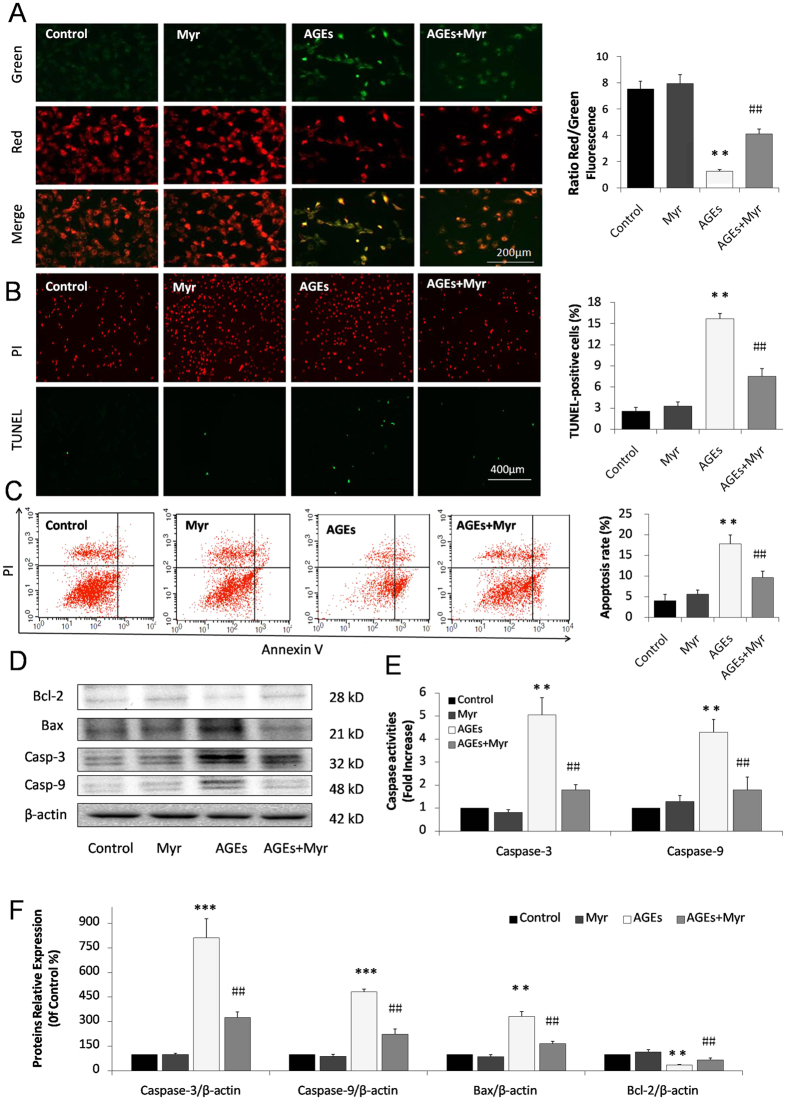
Myr attenuated AGEs-induced mitochondrial injury and apoptosis in H9c2. (**A**) Representative images and bar graphs of JC-1 red/green cells and merges showed that Myr increased the ratio of red to green fluorescence intensity. Representative images and bar graphs of TUNEL-positive nuclei in green fluorescent colour (**B**) and representative images and quantitation of flow cytometry analysis (**C**) showed that Myr inhibited AGEs-induced H9c2 cardiomyocytes apoptosis. (**D**) The expression of apoptosis related proteins in H9c2 cells by immunoblotting analysis. (**F**) The relative expression levels of caspase-3, caspase-9, Bax, and Bcl-2 to β-actin were expressed in the bar graphs. n = 3. (**E**) Caspase-3 and caspase-9 measured by Fluorometric assay. n = 9. **p < 0.01 and ***p < 0.001 vs the control group; ^##^*p* < 0.01 vs AGEs group.

Numerous studies have shown that inflammation and oxidative stress are associated with apoptosis, which is a key factor contributing to DCM^20^,^21^. JC-1 staining was used to detect the disruption of mitochondrial transmembrane potential (MTP), one of the early events of mitochondrial pathway activation of apoptosis. As shown in Fig. 3A, mitochondria in normal H9c2 cells emitted red fluorescence after they were stained by JC-1. AGEs caused an increase in green fluorescence in H9c2 cardiomyocytes, indicating the depolarization of MTP. Myr preconditioning significantly restored MTP and increased the red to green ratio by a wide margin (*p* < 0.01, Fig. 3A). We further performed a TUNEL assay to examine the protective role of Myr in AGEs-induced H9c2 apoptosis. As shown in Fig. 3B, the percentage of TUNEL-positive cells (green) was 15.69% in the AGEs group, and 2.54% in the control group (*p* < 0.01). Pretreatment with Myr significantly reduced the percentage to 7.47% (*p* < 0.01, Fig. 3B). Consistent with the TUNEL assay results, Annexin V/PI staining and quantitative analysis by flow cytometry showed that 17.82 ± 2.2% of H9c2 cells were apoptotic in AGEs treated cells. Myr markedly decreased the percentage of apoptotic cells down to 9.68 ± 1.57% (*p* < 0.01, Fig. 3C). Proteins involved in the mitochondrial pathway of apoptosis were further assayed by western blot analysis. As shown in Fig. 3D, Myr preconditioning reversed the effects of AGEs by decreasing Bcl-2 and increasing Bax expression levels. Similarly, both caspase-3 and caspase-9 were also upregulated in H9c2 cells treated by AGEs, and their expression levels were down-regulated with Myr pretreatment (8.11 vs. 3.44, 4.81 vs. 2.33, respectively, *p* < 0.01, Fig. 3D and F). Additionally, Myr pretreatment significantly inhibited the expression of cleaved caspase-3 and cleaved caspase-9 (Supplementary Figure 1). Consistent with the western blotting results, caspase-3 and caspase-9 activities were significantly higher in the AGEs group than those in the control group. Myr showed inhibitory effects on the activation of these caspase enzymes (*p* < 0.01, Fig. 3E). These results collectively indicate that Myr possesses promising anti-apoptotic effects in cardiomycytes treated by AGEs.

### Myricitrin attenuates AGEs-induced pro-fibrotic and pro-hypertrophic responses in H9c2 cardiomyocytes

**Figure 4:**
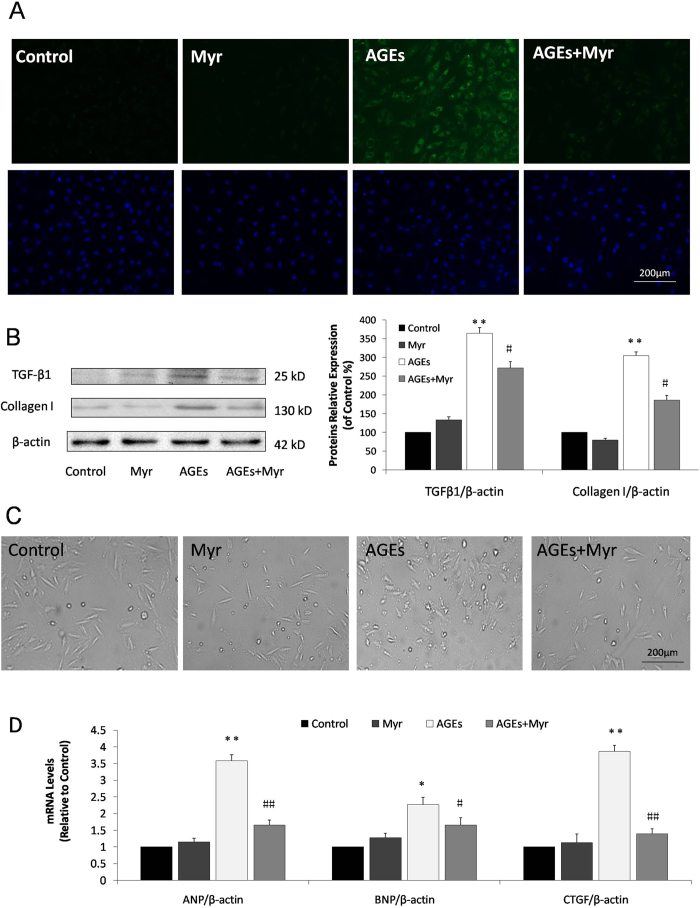
Myr attenuated AGEs-induced fibrosis and hypertrophy in H9c2 cells. H9c2 cells pretreated with Myr for 12 h were incubated with AGEs for 36 h. Immunofluorescence staining for collagen I (**A**) and western blotting for collagen I and TGF-β1 (**B**) in the cells were performed. (**C**) Representative images for cell morphology analysis were obtained by light microscopy. (**D**) Real-time qPCR analysis for pro-hypertrophic gene expression was carried out n = 6. *p < 0.05 and **p < 0.01 vs the control group; ^#^*p* < 0.05 and ^##^*p* < 0.01 vs the AGEs group.

Pro-fibrotic markers were detected in AGEs-induced H9c2 cells pretreated with or without Myr. Collagen I expression was assayed by immunofluorescence staining (Fig. 4A). AGEs resulted in a clearly increased collagen I production in the H9c2 cells. As well, we observed that the expression of the pro-fibrotic proteins, TGF-β1 and collagen I, were significantly increased after 36 h AGEs incubation, as indicated by western blot analysis (Fig. 4B). These alterations were significantly mitigated by pretreatment with Myr (*p* < 0.01, Fig. 4B).

Cardiomyocyte hypertrophy also plays key roles in the pathological development of DCM. The effect of Myr on H9c2 cell hypertrophy was observed using light microscopy. H9c2 cardiomyocytes were pretreated with Myr for 12 h and then incubated with AGEs for 36 h. As shown in Fig. 4C, the AGEs-induced increase in cardiac cell size was clearly suppressed by Myr pretreatment. Subsequently, real-time qPCR was performed to determine the mRNA levels of cardiac hypertrophy-associated transcripts, including ANP, BNP, and CTGF. We observed that these mRNA levels were considerably higher in the AGEs-treated cells compared with those in the control group (3.58, 2.27 and 3.88-fold of the control, respectively, *p* < 0.01), and could be down-regulated by Myr preconditioning (Fig. 4D). Thus, Myr possesses effective anti-fibrotic and anti-hypertrophic activities in H9c2 cells induced by AGEs.

### Myricitrin exerts cardiac protective effects by activating the PI3K/Akt pathway and Nrf2/ARE signaling

**Figure 5:**
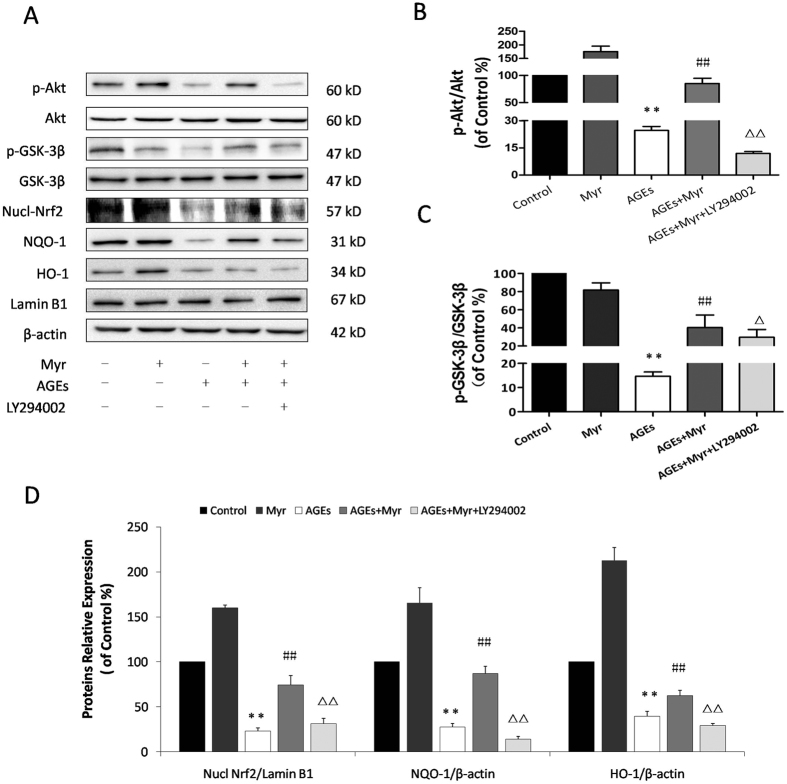
Myricitrin exerts cardioprotective effects by activating the PI3K/Akt pathway and Nrf2/ARE signaling. H9c2 cells were pretreated with the PI3K inhibitor LY294002 (50 μM) for 4 h, and then H9c2 cells pretreated with Myr for 12 h were incubated with AGEs for 36 h. (**A**) The expression of Akt, p-Akt, GSK-3β, p-GSK-3β, and Nrf2 signaling proteins in H9c2 cells by immunoblotting analysis. Quantitative analysis of the p-Akt/Akt expression level (**B**), p-GSK-3β/ GSK-3β expression level (**C**), and the statistical analysis of Nrf2, NQO-1 and HO-1 expression levels relative to the control group (**D**). **p < 0.01 vs the control group; ^##^*p* < 0.01 vs the AGEs group; ^ΔΔ^*p* < 0.01 vs the AGEs + Myr group.

It has been reported that Akt plays key roles in the phosphorylation of Glycogen synthase kinase-3β (GSK-3β), which is an inactive form of GSK-3β. The PI3K inhibitor LY294002 was employed to further confirm the key role of the PI3K/Akt pathway in the protective effects of myricitrin pretreatment. Results from western blotting showed that AGEs significantly suppressed Akt phosphorylation and subsequently inhibited GSK-3β phosphorylation (Fig. 5A–C). Myr pretreatment was found to promote the phosphorylation of Akt and GSK-3β, which could be abolished by Akt inhibitor (LY294002). Furthermore, Nrf2, HO-1 and NQO-1 expression levels were correspondingly reversed (Fig. 5A and D). These results collectively indicate that myricitrin activates Nrf2-mediated anti-oxidant signaling via activation of Akt pathway.

### Myricitrin improves cardiac function and attenuates diabetes-induced cardiac pathological alterations

**Figure 6:**
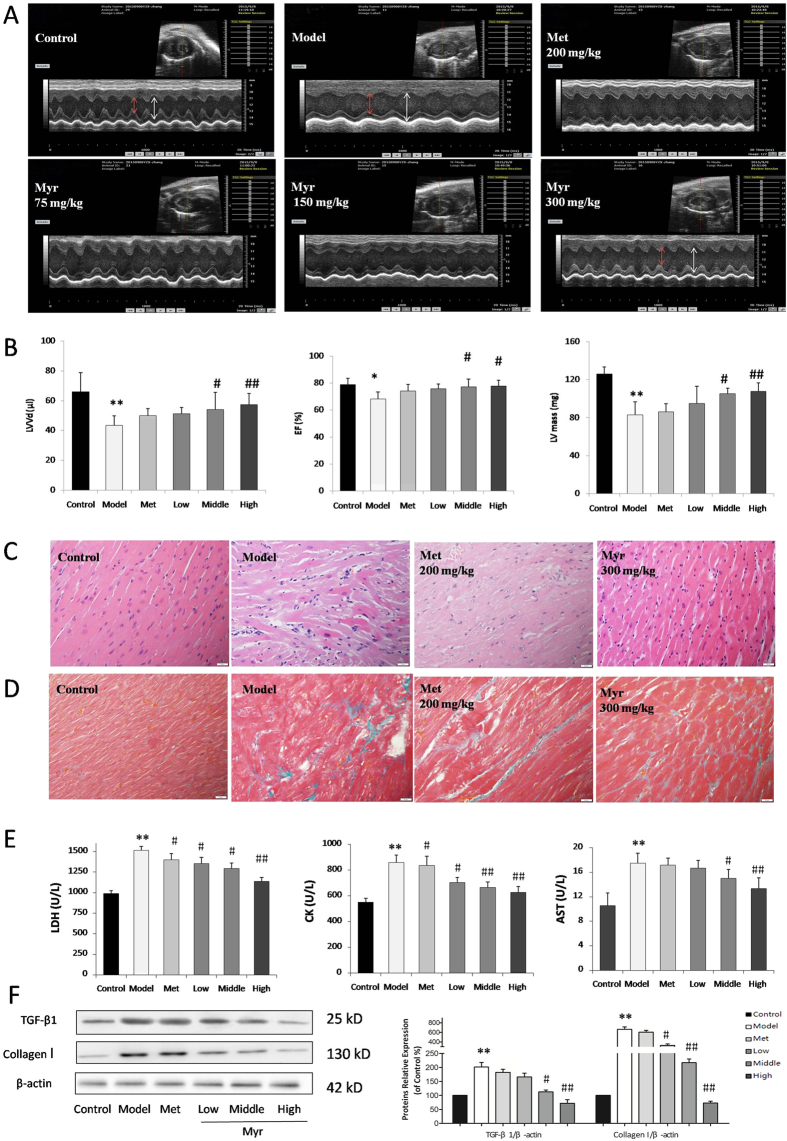
Myr improved cardiac function and attenuated diabetes-induced cardiac pathological alterations. (**A**) Representative images of M-mode echocardiogram. (**B**) Bar diagram showing quantitative data of echocardiography parameters (LVVd, %EF, and LV mass). (**C**,**D**) Representative images of HE and Masson staining of heart tissue. (**E**) The levels of myocardial enzymes (LDH, CK and AST) were determined, n = 6. (**F**) Representative images and bar graphs of collagen I and TGF-β1 by western blot showed that Myr significantly inhibited cardiac fibrosis. n = 3, **p < 0.01 vs the control group; ^#^*p* < 0.05 and ^##^*p* < 0.01 vs the model group.

M-mode echocardiography was performed to assess the cardiac function at 8 weeks after streptozotosin (STZ) injection into mice. LVVd, EF%, and LV mass significantly decreased in diabetic mice compared with a control group, while Myr treatment rescued the attenuated LVVd, EF%, and LV mass in diabetic mice (*p* < 0.05, or *p* < 0.01, Fig. 6A,B). Hemotoxylin and eosin staining of heart tissue showed that myocardial fibers arranged regularly and cardiac myocytes showed normal morphology in the control group (Fig. 6C). However, in the diabetic model group, the arrangement of cardiac fibers was disordered and the cardiac myocytes appeared swollen. Myr treatment ameliorated these structural abnormalities in the hearts of diabetic mice. In comparison, the impact of metformin on diabetic heart was not as potent as Myr. Additionally, Masson staining was performed to investigate cardiac fibrosis resulting from collagen deposition. As is shown in Fig. 6D, conspicuous fibrosis was found in diabetic hearts, with destroyed and disorganized collagen network structure in the interstitial and perivascular areas. In contrast, the fibrotic alterations in the heart were dramatically improved when diabetic mice were treated with 300 mg/kg Myr.

Western blot analysis was employed to determine protein levels of collagen I as well as TGF-β1, which stimulates collagen production. As shown in Fig. 6F, diabetes promoted the expression of both collagen I and TGF-β1 levels, as compared to the control group (6.63 and 2.01-fold of control, respectively, *p* < 0.01). Myr treatment significantly down-regulated the increased collagen I and TGF-β1 expression levels in a dose-dependent manner (*p* < 0.05 or *p* < 0.01). However, the benefit of metformin to these fibrotic proteins was weak. Consistent with the pathological changes, serum lactate dehydrogenase (LDH), creatine kinase (CK), and aspartate transaminase (AST) levels were increased in the diabetic model group compared to those of the control group, and this increase was abrogated by Myr treatment (*p* < 0.05 or *p* < 0.01, Fig. 6E). These results collectively revealed that Myr is able to improve the abnormalities of cardiac function as well as cardiac pathological alterations brought about by long-term high glucose.

### Myricitrin attenuates diabetic myocardial inflammation and promotes the expression of Nrf2-mediated anti-oxidative enzymes

**Figure 7:**
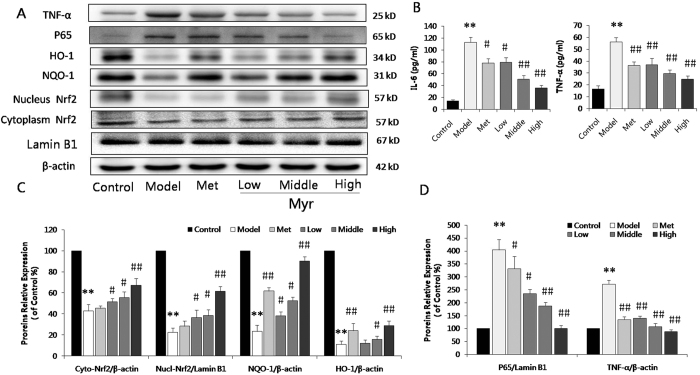
Myr attenuated diabetic myocardial inflammation and promoted expression of Nrf2-mediated anti-oxidative enzymes. (**A**) The expression of P65, TNF-α, and Nrf2 signaling proteins in the diabetic heart by immunoblotting analysis. (**B**) IL-6 and TNF-α levels were determined by ELISA. n = 6. The relative expression levels of Cyto-Nrf2, Nucl-Nrf2, NQO-1, and HO-1 (**C**), as well as P65 and TNF-α relative to β-actin and Lamin B1 (**D**) were expressed in the bar graphs. **p < 0.01 vs the control group; ^#^*p* < 0.05 and ^##^*p* < 0.01 vs the model group.

We further examined the anti-inflammatory and anti-oxidative effects of Myr in diabetic animals. Serum inflammatory cytokines IL-6 and TNF-α were detected using ELISA kits. As shown in Fig. 7B, IL-6 and TNF-α levels in diabetic mice were significantly higher than those in normal mice (112.41 *vs.* 14.36 pg/ml, 56.21 *vs.* 16.35 pg/ml, respectively, *p* < 0.01), which were reduced to 35.72 and 24.83 pg/ml, respectively (300 mg/ml Myr, *p* < 0.01). The effects on TNF-α and P65 expression was subsequently examined by western blot analysis. The increase in levels of TNF-α and P65 in diabetic hearts was attenuated by treatment with Myr in a dose-dependent manner (*p* < 0.05 or *p* < 0.01, Fig. 7A and D). In addition, metformin showed a meliorative effect on inflammatory cytokines and P65 in diabetic mice.

The anti-oxidative enzymes regulated by Nrf2 signaling are key contributors to fight against oxidative stress under pathological conditions. In accordance with our *in vitro* results, Nrf2, HO-1 and NQO-1 were markedly inhibited in diabetic mice compared to the control group (Fig. 7A). This was reversed by Myr treatment in a dose-dependent manner (*p* < 0.05 or *p* < 0.01, Fig. 7A and C). Interestingly, the regulative effect of metformin on Nrf2 was insignificant. However, HO-1 and NQO-1 levels in the met group were significantly higher than those in the diabetic model group. Together, these results indicate that Myr can effectively attenuate inflammation and activate anti-oxidative enzymes through Nrf2 signaling.

### Myricitrin attenuates apoptosis in diabetic myocardiocytes through the activation of Akt signaling and the inhibition of ERK1/2 signaling

**Figure 8:**
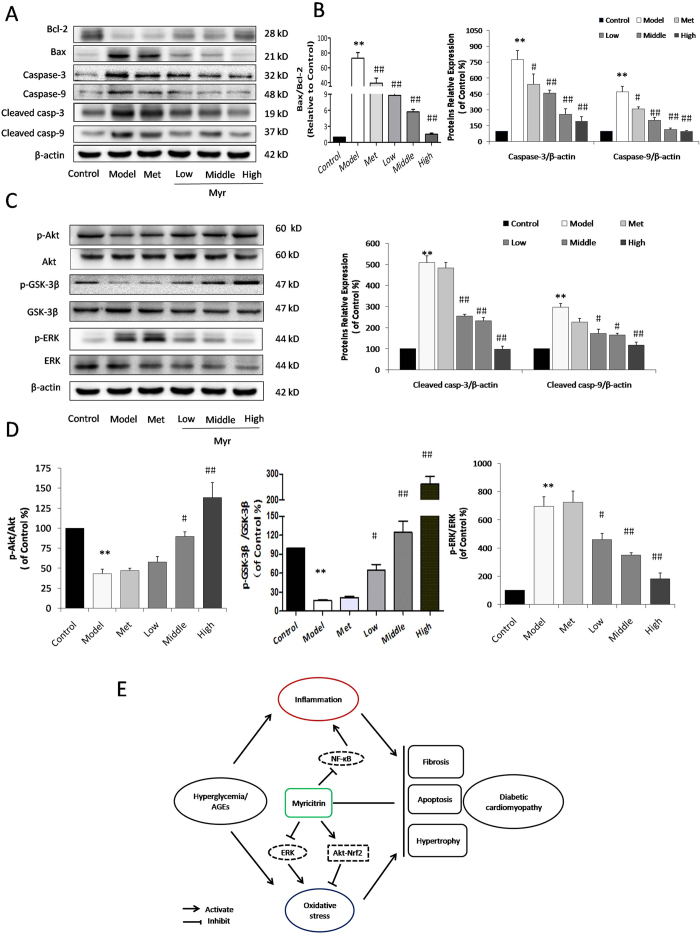
Myr attenuated apoptosis in the diabetic heart through activation of Akt signaling and inhibition of ERK1/2 signaling. (**A**) Representative images of apoptotic proteins and (**B**) bar diagrams showing that Myr effectively prevented diabetes-induced apoptosis. (**C**) The Akt, GSK-3β and ERK phosphorylation were determined by western blotting; densitometric quantification is shown in the bar diagrams (**D**). (**E**) Schematic illustration for the prevention of Myr from diabetes/AGEs-induced injury in cardiomyocytes and diabetic mice. n = 3, **p < 0.01 vs the control group; ^#^*p* < 0.05 and ^##^*p* < 0.01 vs the model group.

To further elucidate the mechanism of action of Myr in the protection of diabetes-induced apoptosis, we examined the levels of Akt, ERK1/2 and apoptotic related proteins by western blot analysis. As shown in Fig. 8A, the dramatic increase in Bax/Bcl-2 ratio, caspase-3, caspase-9, cleaved-caspase-3, and cleaved-caspase-9 expression in diabetic mice was reduced by Myr treatment (*p* < 0.01). The PI3K/Akt and ERK1/2 pathways are involved in multiple cellular processes, including cell growth and survival. Numerous studies indicate that they signal upstream of Nrf2^22^. Results from western blotting showed that phosphorylation of Akt and phosphorylation of ERK1/2 were significantly inhibited and activated in diabetic hearts, respectively (0.43, 6.96-fold of control, respectively, *p* < 0.01, Fig. 8B). Also, the phosphorylation of GSK-3β located at downstream of Akt was inhibited in diabetic hearts (Fig. 8B and D), which was closely to the degradation of Nrf2. Myr treatment was found to enhance the phosphorylation of Akt and GSK-3β and to restrain the phosphorylation of ERK1/2, exerting its anti-oxidative and anti-inflammatory roles in the protection against DCM.

## Discussion

This study has shown, for the first time, that treatment with Myr can significantly protect the heart from the structural and functional alterations that are associated with cardiac inflammation, oxidative stress and apoptosis in STZ-induced diabetic mice. *In vitro* AGEs-induced H9c2 cardiomyocyte injury, characterized by cell fibrosis and hypertrophy accompanied by aggressive inflammation and oxidative stress, could effectively be attenuated by Myr preconditioning. These findings reveal that Myr might be a potential therapeutic agent to fight against diabetic cardiomyopathy.

In recent years, great progress has been made in the treatment of DCM. Continuous high glucose in diabetes results in multiple detrimental outcomes, including the production of inflammatory cytokines and AGEs. Abnormal metabolic function is closely correlated with increased AGEs, which is involved in the pathogenesis of diabetes as well as related complications. It has been well accepted that the overproduction of AGEs in diabetes is a risk factor for DCM^23^,^24^,^25^. In our own *in vitro* experiments, AGEs were confirmed to induce H9c2 cardiomyocyte injury. The mechanism that causes cardiac injury by AGEs is complex and diverse. Inflammatory and oxidative stress responses are the main drivers of pathogenesis in DCM. These conditions are activated by AGEs, which interact with RAGE, a specific receptor on cell surfaces^26^,^27^. AGEs increase the generation of oxygen radicals, which in turn, themselves, activate specific signaling pathways. In particular, this stimulates the nuclear translocation of NF-κB, which plays a pivotal role in the control of inflammatory responses and apoptosis^28^. Consistent with previous literature, in our study, Myr was found to reduce the AGEs/diabetes-induced activation of NF-κB and pro-inflammatory cytokine levels both *in vitro* (Fig. 2A and B) and *in vivo* (Fig. 7A and B), which contributes to its protection against DCM.

An early hallmark of DCM is left ventricular (LV) diastolic dysfunction, which precedes the onset of systolic dysfunction^29^. The pathogenesis of LV dysfunction in the diabetic heart has not been fully clarified. In the present study, STZ injection resulted in morphological alterations to heart tissue, increased interstitial collagen deposition, and up-regulation of TGF-β1, indicating obvious cardiac fibrosis and hypertrophy, which eventually cause the development of DCM. M-mode echocardiography showed that LV end diastolic volume (LVVd) significantly decreased in the diabetic model group compared to the control group, which indicated LV diastolic dysfunction.

Myr treatment significantly attenuated cardiac morphological abnormality, suppressed interstitial collagen deposition, reversed the decreased LV mass, and eventually mitigated cardiac dysfunction (Fig. 6A–E). In line with the pathological changes induced by STZ, Myr reduced the elevated serum LDH, CK, and AST levels. In addition, AGEs induction of H9c2 cell fibrosis and hypertrophy was investigated. We found that AGEs incubation induced H9c2 cell fibrosis and hypertrophy as indicated by the up-regulation of TGF-β1 and collagen I protein levels as well as ANP, BNP and CTGF mRNA levels. These changes were ameliorated by Myr preconditioning (Fig. 4). Interestingly, metformin did not show equal or better cardiac protective effect as Myr. The key point is the mechanistic difference between these two agents. Metformin can specially activate AMPK, subsequently exerting the role in relieving hyperglycemia^30^ and anti-inflammation^31^. In comparison with metformin, Myr not only could ameliorate oxidative stress and inflammation through Akt-dependent activating Nrf2 signaling and inhibiting NF-κB pathway (Fig. 7A, C and D), but could significantly suppressed TGF-β1 signaling mediating the expression of collagen I that was tightly related to cardiac fibrosis.

Chronic high glucose and AGEs in diabetes alter normal homeostasis, resulting in an increase in the generation of ROS^32^,^33^. Treatment with Myr could effectively promote Nrf2 expression and its translocation to the nucleus, which in turn up-regulated HO-1, NQO-1, and γ-GCS to eliminate ROS both *in vitro* (Fig. 2E and F) and *in vivo* (Fig. 7A and C). Inflammation and oxidative stress are directly related to apoptosis in the hearts of diabetic patients and animals^20^,^21^,^34^. Given that Myr ameliorated AGEs-induced oxidative stress, we hypothesized that it subsequently suppressed cardiomyocyte apoptosis. Metabolic disorders of mitochondria induce superfluous ROS generation and are closely associated with apoptosis. In the current study, AGEs incubation resulted in a loss of mitochondrial membrane potential (mtPTP opening), which was restored by pretreatment with Myr. Subsequently, results from TUNEL and Annexin V/PI staining showed that Myr preconditioning could mitigate H9c2 cells apoptosis induced by AGEs (Fig. 3C and D). As Bcl-2 family proteins are main mediators of apoptotic processes, Bax and Bcl-2 are reported to regulate mitochondrial permeability. In accordance with previous studies^35^,^36^, AGEs or hyperglycemia in diabetic mice can increase the Bax/Bcl-2 ratio by generating superfluous ROS, which can decrease mtPTP and subsequently activate caspase-3 expression. Treatment with Myr reduced the expression of pro-apoptotic Bax, and increased expression of the anti-apoptotic Bcl-2, indicating that inhibition of cardiac cell apoptosis may be one of pivotal mechanisms by which Myr attenuates DCM.

The serine/threonine kinase Akt is an important component of the intracellular signaling pathways tightly linked with apoptosis, insulin resistance, and acute myocardial ischemia/reperfusion injury^37^,^38^,^39^. Akt signaling can impact on crosstalk with other signaling pathways including MAPK signaling, and NF-κB signaling pathways. Additionally, the ERK signaling pathway is reported to be involved in DCM, wherein ERK is significantly phosphorylated and inhibits Nrf2 signaling, resulting in cell hypertrophy^12^,^40^. Furthermore, our previous studies have confirmed that phosphorylation of Akt regulates Nrf2 activation and HO-1 expression^16^,^41^. However, the impacts of Myr on Akt and ERK signaling in the diabetic heart were unclear. As we hypothesized, not only was phospho-Akt inhibited, but phospho-ERK was activated in diabetic animals, both of which could be reversed by Myr in a dose-dependent manner (Fig. 8B). Increasing evidence has revealed a novel mechanism of Nrf2 regulation that is independent of keap1. Chowdhry *et al*. found that Nrf2 is controlled by two distinct β-TrCP recognition motifs in its Neh6 domain, one of which can be modulated by GSK-3 activity. Nrf2 contains two motif, including DSAPGS and DSGIS, and the later could be phosphorylated by GSK-3^42^. Rada *et al*. also found that SCF/β-TrCP (DSGIS) promotes GSK-3-dependent degradation of the Nrf2 transcription factor in a Keap1-independent manner, indicating that activated GSK-3 phosphorylated Nrf2 in the Neh6 domain to promote recognition of Nrf2 by β-TrCP and subsequent protein degradation^43^. AGEs could inhibit Akt activation and subsequently facilitated functional GSK-3 to result in degradation of Nrf2. Both the activation of Akt and phosphorylation of GSK-3β by Myr in turn regulate Nrf2 signaling to attenuate oxidative stress in DCM.

The relationship between cardiovascular disease and diabetes is complex and is assciated with multiple facts. This research mainly focuses on observing the cardioprotective effects of Myr on oxidative stress and inflammation induced by superfluous AGEs and hyperglycemia. However, Recent researches have reported that calcium release channel controls insulin release and glucose homestasis, which is tightly related to diabetic cardiovascular diseases and heart failure^44^,^45^,^46^,^47^,^48^. Thus, it would be interesting to investigate whether Myr has a cardioprotective effect through regulation of calcium release channel. Another limitation is related to the animal models. Type 2 diabetic animals such as db/db or ob/ob mice are also suitable for modeling DCM. Furthermore, the protective effects of Myr in Nrf2-knockout H9c2 cardiomyocytes is unknown. Finally, how Akt and ERK signaling affects each other is not fully understood. Thus, our future studies will address these questions.

In summary, we have shown that Myr possesses cardioprotective effects against DCM in an STZ-induced mouse model. This occurs via the activation of Akt and inhibition of ERK signaling, and the suppression of cardiac oxidative stress, inflammation, and apoptosis. Also, we have demonstrated that Myr can attenuate AGEs-induced H9c2 fibrosis and hypertrophy. Accordingly, Myr should be considered as a potential therapeutic medicine to prevent diabetic cardiomyopthy.

## Methods

### Animals

The BALB/c mice (male, 6–8 weeks old) used in this study were purchased from Vital River Laboratory Animal Technology (Beijing, China). The mice were maintained under standard laboratory conditions (room temperature at 22 °C, humidity of 60% with a 12 h light/dark cycle) and fed with a standard pellet diet and water *ad libitum*. All of the procedures were performed in accordance with the guidelines of the Experimental Laboratory Animal Committee of Chinese Academy of Medical Sciences and Peking Union Medical College and the principles and guidelines of the National Institutes of Health Guide for the Care and Use of Laboratory Animals published by the US National Institutes of Health (NIH Publication No. 85–23, revised 1996), and approved by the Experimental Laboratory Animal Committee of Chinese Academy of Medical Sciences and Peking Union Medical College. All sacrifices were performed under pentobarbitone anesthesia, and every effort was made to minimize animal suffering.

After one week adaptation, DM was induced by a single intraperitoneal (i.p.) injection of STZ (Sigma Chemicals, St. Louis, MO) at the dose of 150 mg/kg dissolved in 100 mM citrate buffer (pH 4.5). Control animals received intraperitoneal injection of citrate buffer. One week later, tail blood glucose levels were measured using a glucometer (Roche). Mice with fasting-blood glucose >12 mmol/L were considered diabetic and were used for the further experiment. The mice were randomly divided into 6 groups: (1) control group (Control); (2) diabetic model group (DM, Model); (3) DM + metformin 200 mg/kg/day group (Met); (4) DM + Myr 75 mg/kg/day group (Low); (5) DM + Myr 150 mg/kg/day group (Middle); (6) DM + Myr 300 mg/kg/day group (High). Drinking water with aseptic carboxymethyl cellulose sodium (CMC-Na, 0.5%) was used as the vehicle to dissolve the powders. Myricitrin or metformin was fed to the mice by gavage every day for 8 weeks. The control and model groups received the aseptic 0.5% CMC-Na treatment every day (i.g.). Doses of myricitrin and metformin used were based on the previous studies 41,49.

### Echocardiographic measurements

M-mode echocardiography was performed using Vevo 770™ High Resolution Imaging System (VisualSonics Inc, Canada) as previously described^50^. The mice were anaesthetized with abdominal injection of avertin (2,2,2-tribromoethanol, prepared as a 1.2% solution and used in mice at a dosage of 0.2 ml/10 g body weight). The chests of the mice were shaved, which were then placed in the recumbent position. LV end-diastolic volume (LVVd), ejection fraction (EF), and LV Mass (AW) were automatically calculated by the ultrasound machine.

### Heart Histopathological Examination

At the end of the experiment, hearts of the mice were excised, the left ventricles were fixed in 4% buffered paraformaldehyde for more than 48 h and embedded in paraffin blocks, sectioned, stained with hematoxylin and eosin or Masson stain, and examined using a light microscope by a pathologist who was blinded to the groups under study.

### Measurement of myocardial enzymes activities

Serum myocardial enzyme activities of LDH, CK and AST were measured with corresponding detection kits according to the manufacturer’s instructions (Nanjing Jiancheng Bioengineering, China).

### ELISA for detection of IL-6 and TNF-α levels

Blood samples were collected from the eye socket vein of the mice after eight weeks of drug treatment. IL-6 and TNF-α levels in the blood were determined by ELISA kit (Biolegend lnc.) according to the manufacturer’s recommendations. OD value was detected using a microplate reader at 450 nm (MQX 200, BioTek Instruments, Winooski, VT, USA).

### Real-time qPCR

TRIzol Reagent was used to extract total RNA. RNA concentrations and purities were quantified using spectrophotometer (Nanodrop 2000c, Thermo Fisher). cDNA was synthesized using PrimeScript master mix reagent Kit. Then Real-time PCR was carried out using SYBR Premix Ex Taq reagent Kit (Takara Biotechnology (Dalian) Co., Ltd.) in a Bio-Rad C1000 detecting system (Bio-Rad Laboratories, Hercules, CA, USA). Primers used for real-time RT-PCR were as follows: mouse ANP sense, 5′-GTGCGGTGTCCAACACAGAT-3′ and antisense, 5′-TCCAATCCTGTCAATCCTACCC-3′; mouse BNP sense, 5′-CTGAAGGTGCTGTCCCAGAT-3′ and antisense, 5′-GTTCTTTTGTGAGGCCTTGG-3′; mouse CTGF sense, 5′-ACTATGATGCGAGCCAACTGC-3′ and antisense, 5′-TGTCCGGATGCACTTTTTGC-3′; mouse β-actin sense, 5′-TGCACCACCAACTGCTTAG -3′ and antisense, 5′-GGATGCAGGGATGATGTTC-3′. β-actin was used as control of mRNA expression. The fold change for all the samples was calculated by2^−△△Ct^ methods.

### Materials

Myricitrin was isolated from the grounded barks of *Myriace rubrae* and was identified by Professor Rui-le Pan. The purity is over 99%, as detected by high-performance liquid chromatography (UV and DAD)^41^. Metformin (Sino-American Shanghai Squibb Pharmaceuticals Ltd,) was used as the positive control in this study. All cell culture materials, Dulbecco’s modified Eagle’s medium (DMEM), fetal bovine serum (FBS), and penicillin/streptomycin were obtained from Gibco (NY, USA). The kits for determining the CK, LDH and AST enzyme levels were purchased from Jiancheng Bioengineering Institute (Nanjing, China). Akt inhibitor (LY294002) was purchased from Selleckchem (TX, USA). Primary antibodies against Akt, p-Akt, GSK-3β, p- GSK-3β, P65, p-IKKβ, TNF-α, ERK, p-ERK, TGF-β1, Collagen I, Caspase-3, Caspase-9, Bcl-2, Bax, Nrf2, HO-1, NQO-1, γ-GCS, Lamin B1 and β-actin were obtained from Santa Cruz Biotechnology (CA, USA). FITC-anti-rabbit IgG second antibody was purchased from Molecular Probes (Life Technologies, CA, USA).

### Preparation of AGE-BSA

AGE-BSA was prepared according to the protocol of Zhang *et al*.^51^. Briefly, 0.07 g bovine serum albumin in PBS was incubated with 0.7926 g D-glucose at 37 °C for eight weeks. Control albumin was incubated without glucose. Endotoxin was removed by Pierce endotoxin removing gel and was determined by ToxinSensor™ chromogenic LAL Endotoxin Assay Kit (GenScript, Piscataway, NJ, USA), which was less than 500  U/L.

### Cell culture and treatment

Rat embryonic cardiomyoblast-derived H9c2 cardiomyocytes were purchased from the Cell Bank of the Chinese Academy of Sciences (Shanghai, China) and cultured in DMEM (Gibco, glucose content, 5.5 mM), supplemented with 10% fetal bovine serum, 1% penicillin/streptomycin in a 5% CO2 atmosphere at 37 °C. For all experiments, the cells were plated at an appropriate density according to the experimental design and were grown for 24 h to reach 70–80% confluence before experimentation. The cells were divided into following groups: (1) BSA group (Control); (2) BSA + Myiricitrin group (Myr); (3) AGE-BSA group (AGEs); (4) AGE-BSA + Myricitrin (AGEs + Myr). H9c2 cells were places in 96-well places at 8 × 10^3^ per well for 24 h. The cells first treated for 12, 24 and 36 h with various concentrations of AGEs.

### Cell viability and morphological analysis

MTT assay was employed to determine the cell viability of the H9c2 cardiomyocytes. Cells cultured in 96-well plates (8 × 10^3^ cells/well) were incubated with MTT solution (1 mg/ml final concentration) at 37 °C for 4 h after the different treatments. The formazan crystals were dissolved with dimethyl sulfoxide (DMSO, 150 μl/well), and the absorbance was detected at 570 nm on a microplate reader. Cell viability was expressed as the percentage of MTT reduction compared with the control conditions. To visualize the morphological change, after treatment H9c2 cells were fixed with 4% paraformaldehyde ans observed using a light microscope (200 × amplification, Nikon, Japan).

### Detection of Mitochondrial Superoxide (ROS)

MitoSOX Red (Molecular Probes), a mitochondrial superoxide indicator, was employed to detect mitochondrial superoxide production in H9c2 cells as described previously^52^. Briefly, after treatment, the cells were washed once with PBS and incubated with MitoSOX Red (5 μM) in the dark at 37 °C for 10 min. The cells were washed with PBS and then observed with a fluorescence microscope. The fluorescence of MitoSOX Red was detected on a microplate reader at the excitation and emission wavelengths of 510 nm and 580 nm, respectively. As well, the fluorescence was analyzed by flow cytometry (BD Biosciences, USA).

### Mitochondrial transmembrane potential (∆Ψm)

JC-1 (Invitrogen, Waltham, MA, USA) was used to determine the changes in mitochondrial transmembrane potential as previous reported^19^. Briefly, after treatment, H9c2 cardiomyocytes were incubated with JC-1 (5 μmol/L) in the dark at 37 °C for 30 min and were then washed with PBS followed by fluorescence microscopy (DM4000B, Leica, Germany).

### Terminal Deoxynucleotidyl Transferase-mediated dUTP Nick End Labelling (TUNEL) Staining

Apoptotic H9c2 cardiomyocytes were visualized by TUNEL staining according to the manufacturer’s instructions (Biovision, Milpitas, CA, USA). Briefly, H9c2 cells were cultured in 24-well plates for 24 h. After treatment, the cells were fixed with 1% paraformaldehyde for 15 min. After twice washes with PBS, incubated the cells in the DNA labeling solution for 60 min at 37 °C, and then incubated the cells with anti-BrdU-FITC antibody solution in the dark for 30 min. Images were captured using a fluorescence microscope, and the apoptotic cells were counted with at least 100 cells from five randomly selected fields in each group.

### Flow cytometric detection of apoptosis

After the cardiomyocytes cells were treated with AGEs, apoptosis was determined by Annexin V-FITC/PI Apoptosis kit according to the manufacturer’s instructions (Invitrogen, USA). In brief, the H9c2 cells were harvested, washed twice with cold PBS, incubated with the 5 μl FITC-Annexin V and 1 μl PI working solution (100 μg/ml) for 30 min in the dark at room temperature, and cellular fluorescence was measured by flow cytometry analysis.

### Analysis of caspase-3 and caspase-9 activities

Caspase-3 and caspase-9 activities were evaluated with Fluorometric Assay Kits (BioVision, USA) according to the manufacturer’s instructions respectively. Briefly, the cells were resuspended in lysis buffer and kept on ice for 10 min. Then, 50 μl of 2X reaction buffer containing 10 mM dithiothreitol was added to each sample. 5 μl of 1 mM substrate (DEVD-AFC or LEHD-AFC for caspase-3 or caspase-9, respectively) was added and incubated at 37 °C for 1.5 h. The samples were read on a fluorometer at 400 nm excitation and 505 nm emission wavelengths. The fold-increases in caspase activities were determined by comparing the results with the level of the control group.

### Western blot assay

Cytoplasmic and nuclear protein samples were separated by protein extraction kits containing 1% phenylmethylsulfonyl fluoride (CoWin Bioscience Co., Ltd., Beijing, China).Western blots were performed using a standard blotting protocol, as described previously^53^. Equal amounts (10 μg) of protein fractions were separated by electrophoresis on 10% SDS-PAGE gels, and transferred into nitrocellulose membranes. Antibody binding to the membranes was visualized by enhanced chemiluminescence.

### Immunofluorescence assay for collagen I

After treatment, H9c2 cells were fixed in 4% paraformaldehyde for 10 min, permeabilized with 0.1% Triton X-100 for 5 min, and blocked with BSA for 30 min at room temperature. Then cells were washed twice with PBS and incubated primary antibody for collagen I overnight at 4 °C, followed by FITC-anti-rabbit IgG second antibody and DAPI for 50 min. Fluorescent images were taken using a Leica DM 4000B fluorescent microscope.

### Statistical analysis

Data from at least three independent experiments were expressed as the means ± SD. Statistical comparisons between different groups were measured by one-way ANOVA followed by the Student-Newman-Keuls test. The level of significance was set at *p* < 0.05.

## Additional Information

**How to cite this article:** Zhang, B. *et al*. Myricitrin Alleviates Oxidative Stress-induced Inflammation and Apoptosis and Protects Mice against Diabetic Cardiomyopathy. *Sci. Rep.* **7**, 44239; doi: 10.1038/srep44239 (2017).

**Publisher's note:** Springer Nature remains neutral with regard to jurisdictional claims in published maps and institutional affiliations.

## Supplementary Material

Supplementary Figure 1

## References

[b1] International Diabetes Federation. Diabetes Atlas 7th Edition http://www.diabetesatlas.org/2015/12/01 (2015)

[b2] Chavali V., Tyagi S. C. & Mishra P. K. Predictors and prevention of diabetic cardiomyopathy. Diabetes Metab Syndr Obes 6, 151–60 (2013). 23610527 10.2147/DMSO.S30968 PMC3629872

[b3] Liu F. . Upregulation of MG53 induces diabetic cardiomyopathy through transcriptional activation of peroxisome proliferation-activated receptor alpha. Circulation 131, 795–804 (2015). 25637627 10.1161/CIRCULATIONAHA.114.012285

[b4] Cai L. . Attenuation by metallothionein of early cardiac cell death via suppression of mitochondrial oxidative stress results in a prevention of diabetic cardiomyopathy. J Am Coll Cardiol 48, 1688–97 (2006). 17045908 10.1016/j.jacc.2006.07.022

[b5] Vulesevic B. . Methylglyoxal-Induced Endothelial Cell Loss and Inflammation Contribute to the Development of Diabetic Cardiomyopathy. Diabetes 65, 1699–713 (2016). 26956489 10.2337/db15-0568 PMC4878427

[b6] Cheng Y. S., Dai D. Z., Dai Y., Zhu D. D. & Liu B. C. Exogenous hydrogen sulphide ameliorates diabetic cardiomyopathy in rats by reversing disordered calcium-handling system in sarcoplasmic reticulum. J Pharm Pharmacol 68, 379–88 (2016). 26968978 10.1111/jphp.12517

[b7] Montaigne D. . Myocardial contractile dysfunction is associated with impaired mitochondrial function and dynamics in type 2 diabetic but not in obese patients. Circulation 130, 554–564 (2014). 24928681 10.1161/CIRCULATIONAHA.113.008476

[b8] Mulvihill E. E. . Inhibition of Dipeptidyl Peptidase-4 Impairs Ventricular Function and Promotes Cardiac Fibrosis in High Fat-Fed Diabetic Mice. Diabetes 65, 742–54 (2016). 26672095 10.2337/db15-1224

[b9] Ma H. . Advanced glycation endproduct (AGE) accumulation and AGE receptor (RAGE) up-regulation contribute to the onset of diabetic cardiomyopathy. J Cell Mol Med 13, 1751–1764 (2009). 19602045 10.1111/j.1582-4934.2008.00547.x PMC2829341

[b10] Liu M. . NaoXinTong Inhibits the Development of Diabetic Retinopathy in db/db Mice. Evid Based Complement Alternat Med 2015, 242517 (2015). 25821481 10.1155/2015/242517 PMC4363582

[b11] Thornalley P. J. Glycation free adduct accumulation in renal disease: the new AGE. Pediatr nephrolo 20, 1515–1522 (2005). 10.1007/s00467-005-2011-9 16133053

[b12] Ko S. Y., Chang S. S., Lin I. H. & Chen H. I. Suppression of antioxidant Nrf-2 and downstream pathway in H9c2 cells by advanced glycation end products (AGEs) via ERK phosphorylation. Biochimie 118, 8–14 (2015). 26212730 10.1016/j.biochi.2015.07.019

[b13] Yang D. H. . Increased levels of circulating advanced glycation end-products in menopausal women with osteoporosis. Int J Med Sci 11, 453–460 (2014). 24688308 10.7150/ijms.8172 PMC3970097

[b14] Chawla D. . Role of advanced glycation end product (AGE)-induced receptor (RAGE) expression in diabetic vascular complications. Microvasc Res 95, 1–6 (2014). 24984291 10.1016/j.mvr.2014.06.010

[b15] Zhong P. . Blockage of ROS and NF-kappaB-mediated inflammation by a new chalcone L6H9 protects cardiomyocytes from hyperglycemia-induced injuries. Biochim Biophys Acta 1852, 1230–41 (2015). 25736300 10.1016/j.bbadis.2015.02.011

[b16] Zhang B. . Myricitrin Attenuates High Glucose-Induced Apoptosis through Activating Akt-Nrf2 Signaling in H9c2 Cardiomyocytes. Molecules 21 (2016). 10.3390/molecules21070880 PMC6274128 27399653

[b17] Domitrovic R. . Myricitrin exhibits antioxidant, anti-inflammatory and antifibrotic activity in carbon tetrachloride-intoxicated mice. Chem Biol Interact 230, 21–9 (2015). 25656916 10.1016/j.cbi.2015.01.030

[b18] Jin X. . Pioglitazone alleviates inflammation in diabetic mice fed a high-fat diet via inhibiting advanced glycation end-product-induced classical macrophage activation. FEBS J 283, 2295–308 (2016). 27062545 10.1111/febs.13735

[b19] Rani N. . Chrysin, a PPAR-gamma agonist improves myocardial injury in diabetic rats through inhibiting AGE-RAGE mediated oxidative stress and inflammation. Chem Biol Interact 250, 59–67 (2016). 26972669 10.1016/j.cbi.2016.03.015

[b20] Qin W. D. . Poly(ADP-ribose) polymerase 1 inhibition protects cardiomyocytes from inflammation and apoptosis in diabetic cardiomyopathy. Oncotarget 7, 35618–35631 (2016). 27027354 10.18632/oncotarget.8343 PMC5094949

[b21] Yu M. . Inhibiting microRNA-144 abates oxidative stress and reduces apoptosis in hearts of streptozotocin-induced diabetic mice. Cardiovasc Pathol 24, 375–81 (2015). 26164195 10.1016/j.carpath.2015.06.003

[b22] Lee D. S. & Jeong G. S. Butein provides neuroprotective and anti-neuroinflammatory effects through Nrf2/ARE-dependent haem oxygenase 1 expression by activating the PI3K/Akt pathway. Br J Pharmacol 173, 2894–909 (2016). 27465039 10.1111/bph.13569 PMC5055139

[b23] Hou J. . Mangiferin suppressed advanced glycation end products (AGEs) through NF-kappaB deactivation and displayed anti-inflammatory effects in streptozotocin and high fat diet-diabetic cardiomyopathy rats. Can J Physiol Pharmacol, 1–9 (2015). 10.1139/cjpp-2015-0073 26751764

[b24] Guo Z., Huang D., Tang X., Han J. & Li J. Correlation between advanced glycation end-products and the expression of fatty inflammatory factors in type II diabetic cardiomyopathy. Bosn J Basic Med Sci 15, 15–9 (2015). 10.17305/bjbms.2015.619 PMC4690436 26614846

[b25] Yuan Q. . Advanced glycation end-products impair Na( + )/K( + )-ATPase activity in diabetic cardiomyopathy: role of the adenosine monophosphate-activated protein kinase/sirtuin 1 pathway. Clin Exp Pharmacol Physiol 41, 127–33 (2014). 24341361 10.1111/1440-1681.12194

[b26] Liu Y. . AGEs increased migration and inflammatory responses of adventitial fibroblasts via RAGE, MAPK and NF-κB pathways. Atherosclerosis 208, 34–42 (2010). 19959167 10.1016/j.atherosclerosis.2009.06.007

[b27] Mattiello-Sverzut A. C., Petersen S. G., Kjaer M. & Mackey A. L. Morphological adaptation of muscle collagen and receptor of advanced glycation end product (RAGE) in osteoarthritis patients with 12 weeks of resistance training: influence of anti-inflammatory or glucosamine treatment. Rheumatol Int 33, 2215–24 (2013). 23443332 10.1007/s00296-013-2698-z

[b28] Lorenzo O. . Potential role of nuclear factor kappaB in diabetic cardiomyopathy. Mediators Inflamm 2011, 652097 (2011). 21772665 10.1155/2011/652097 PMC3136091

[b29] Schannwell C. M., Schneppenheim M., Perings S., Plehn G. & Strauer B. E. Left ventricular diastolic dysfunction as an early manifestation of diabetic cardiomyopathy. Cardiology 98, 33–9 (2002). 12373045 10.1159/000064682

[b30] Zhou Z. Y. . Metformin exerts glucose-lowering action in high-fat fed mice via attenuating endotoxemia and enhancing insulin signaling. Acta Pharmacol Sin 37, 1063–1075 (2016). 27180982 10.1038/aps.2016.21 PMC4973377

[b31] Zhou Z. . Metformin Inhibits Advanced Glycation End Products-Induced Inflammatory Response in Murine Macrophages Partly through AMPK Activation and RAGE/NFkappaB Pathway Suppression. J Diabetes Res 2016, 4847812 (2016). 27761470 10.1155/2016/4847812 PMC5059570

[b32] Zhao Y. T., Qi Y. W., Hu C. Y., Chen S. H. & Liu Y. Advanced glycation end products inhibit testosterone secretion by rat Leydig cells by inducing oxidative stress and endoplasmic reticulum stress. Int J Mol Med 38, 659–65 (2016). 27315604 10.3892/ijmm.2016.2645

[b33] Ma C. T. . Pepino polyphenolic extract improved oxidative, inflammatory and glycative stress in the sciatic nerves of diabetic mice. Food Funct 7, 1111–21 (2016) 26791916 10.1039/c5fo01358e

[b34] Li W. . EGFR Inhibition Blocks Palmitic Acid-induced inflammation in cardiomyocytes and Prevents Hyperlipidemia-induced Cardiac Injury in Mice. Sci Rep 6, 24580 (2016). 27087279 10.1038/srep24580 PMC5263857

[b35] Hosseinzadeh L. . Curcumin potentiates doxorubicin-induced apoptosis in H9c2 cardiac muscle cells through generation of reactive oxygen species. Food Chem Toxicol 49, 1102–9 (2011). 21295102 10.1016/j.fct.2011.01.021

[b36] Liu Y. . Prolyl hydroxylase 3 interacts with Bcl-2 to regulate doxorubicin-induced apoptosis in H9c2 cells. Biochem Biophys Res Commun 401, 231–7 (2010). 20849813 10.1016/j.bbrc.2010.09.037

[b37] Maurya A. K. & Vinayak M. PI-103 attenuates PI3K-AKT signaling and induces apoptosis in murineT-cell lymphoma. Leukemia & lymphoma, 1–9 (2016). 10.1080/10428194.2016.1225207 27658642

[b38] Li P. . LTB4 promotes insulin resistance in obese mice by acting on macrophages, hepatocytes and myocytes. Nat Med 21, 239–47 (2015). 25706874 10.1038/nm.3800 PMC4429798

[b39] Kong Q. . HSPA12B Attenuated Acute Myocardial Ischemia/reperfusion Injury via Maintaining Endothelial Integrity in a PI3K/Akt/mTOR-dependent Mechanism. Sci Rep 6, 33636 (2016). 27644317 10.1038/srep33636 PMC5028890

[b40] Tan Y. . Diabetic downregulation of Nrf2 activity via ERK contributes to oxidative stress-induced insulin resistance in cardiac cells *in vitro* and *in vivo*. Diabetes 60, 625–33 (2011). 21270272 10.2337/db10-1164 PMC3028364

[b41] Qin M. . Myricitrin attenuates endothelial cell apoptosis to prevent atherosclerosis: An insight into PI3K/Akt activation and STAT3 signaling pathways. Vascul Pharmacol 70, 23–34 (2015). 25849952 10.1016/j.vph.2015.03.002

[b42] Chowdhry S. . Nrf2 is controlled by two distinct β-TrCP recognition motifs in its Neh6 domain, one of which can be modulated by GSK-3 activity. Oncogene 32, 3765–3781 (2013). 22964642 10.1038/onc.2012.388 PMC3522573

[b43] Rada P. . SCF/β-TrCP Promotes Glycogen Synthase Kinase 3-Dependent Degradation of the Nrf2 Transcription Factor in a Keap1-Independent Manner. Mol Cell Biol 31, 1121–1133(2011). 21245377 10.1128/MCB.01204-10 PMC3067901

[b44] Noguchi N. . FKBP12.6 disruption impairs glucose-induced insulin secretion. Biochem Biophys Res Commun 371, 735–740 (2008). 18466757 10.1016/j.bbrc.2008.04.142

[b45] Santulli G. . Calcium release channel RyR2 regulates insulin release and glucose homeostasis. J Clin Invest 125, 1968–1978 (2015). 25844899 10.1172/JCI79273 PMC4463204

[b46] Yaras N. . Effects of diabetes on ryanodine receptor Ca release channel (RyR2) and Ca2 + homeostasis in rat heart. Diabetes 54, 3082–8 (2005). 16249429 10.2337/diabetes.54.11.3082

[b47] Bidasee K. R. . Chronic Diabetes Increases Advanced Glycation End Products on Cardiac Ryanodine Receptors/Calcium-Release Channels. Diabetes 52, 1825–1836 (2003). 12829653 10.2337/diabetes.52.7.1825

[b48] Marks A. R. Calcium cycling proteins and heart failure: mechanisms and therapeutics. J Clin Invest 123, 46–52 (2013). 23281409 10.1172/JCI62834 PMC3533269

[b49] Saeedi Saravi S. S., Hasanvand A., Shahkarami K. & Dehpour A. R. The protective potential of metformin against acetaminophen-induced hepatotoxicity in BALB/C mice. Pharm Biol, 1–8 (2016). 10.1080/13880209.2016.1185633 27252117

[b50] Gao X. . Allopurinol attenuates left ventricular dysfunction in rats with early stages of streptozotocin-induced diabetes. Diabetes Metab Res Rev 28, 409–17 (2012) 22389139 10.1002/dmrr.2295

[b51] Zhang W. . Role of Src in Vascular Hyperpermeability Induced by Advanced Glycation End Products. Sci Rep 5, 14090 (2015). 26381822 10.1038/srep14090 PMC4585381

[b52] Xie W. . Mitochondrial oxidative stress promotes atrial fibrillation. Sci Rep 5, 11427 (2015). 26169582 10.1038/srep11427 PMC4501003

[b53] Yu Y. . Cardioprotective effects of Notoginsenoside R1 against ischemia/reperfusion injuries by regulating oxidative stress- and endoplasmic reticulum stress- related signaling pathways. Sci Rep 6, 21730 (2016). 26888485 10.1038/srep21730 PMC4757886

