# Supplementary material for: Myricitrin Alleviates Oxidative Stress-induced Inflammation and Apoptosis and Protects Mice against Diabetic Cardiomyopathy
Source: Sci Rep. 2017 Mar 13;7:44239. doi: 10.1038/srep44239 (PMC5347164; doi:10.1038/srep44239)
Supplement: Supplementary Figure 1 [file srep44239-s1.pdf]

Myricitrin Alleviates Oxidative Stress-induced Inflammation and Apoptosis and Protects Mice against Diabetic Cardiomyopathy

Bin Zhang[1,2,3,4], Qiang Shen [5], Yaping Chen [6], Ruile Pan [1], Shihuan Kuang [7], Guiyan Liu [6,*] Guibo Sun [1,2,3,4,*] & Xiaobo Sun [1,2,3,4,*]

[1] Institute of Medicinal Plant Development, Peking Union Medical College and Chinese Academy of Medical Sciences, Beijing 100193, China
[2] Key Laboratory of Bioactive Substances and Resources Utilization of Chinese Herbal Medicine, Ministry of Education, Beijing 100193, China
[3] Beijing Key Laboratory of Innovative Drug Discovery of Traditional Chinese Medicine (Natural Medicine) and Translational Medicine, Beijing 100193, China
[4] Key Laboratory of efficacy evaluation of Chinese Medicine against glyeolipid metabolism disorder disease, State Administration of Traditional Chinese Medicine, Beijing 100193, China
[5] Center of Research and Development on Life Sciences and Environmental Sciences, Harbin University of Commerce, Harbin 150076, China
[6] School of Life Science, Beijing Institute of Technology, Beijing 100081, China
[7] Department of Animal Sciences, Purdue University, West Lafayette, IN 47907, USA

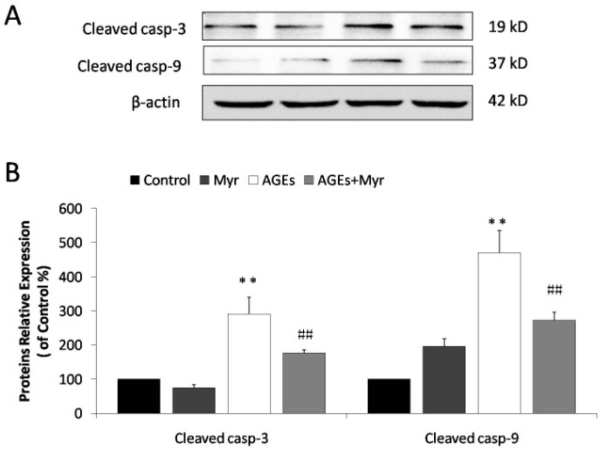

Supplementary Figure 1. Myr attenuated AGEs-induced apoptosis in H9c2 cells. H9c2 cells pretreated with Myr for 12 h were incubated with AGEs for 36 h. (A) Representative images of cleaved caspase-3 and cleaved caspase-9 and (B) bar diagrams showing that Myr effectively prevented AGEs-induced apoptosis. ***p* < 0.01 vs the control group; ##*p* < 0.01 vs the AGEs group.
